# One-pot synthesis of α-Fe_2_O_3_ nanospheres by solvothermal method

**DOI:** 10.1186/1556-276X-8-213

**Published:** 2013-05-06

**Authors:** Caihua Wang, Yumin Cui, Kaibin Tang

**Affiliations:** 1 School of Chemistry and Chemical Engineering, Fuyang Normal College, Fuyang, 236041, People's Republic of China; 2 Anhui Province Key Laboratory for Degradation and Monitoring of Pollution of the Environment, Fuyang, 236041, People's Republic of China; 3 Department of Chemistry, University of Science and Technology of China, Hefei, 230026, People's Republic of China

**Keywords:** Solvothermal method, Fe_2_O_3_, Nanospheres

## Abstract

We have successfully prepared α-Fe_2_O_3_ nanospheres by solvothermal method using 2-butanone and water mixture solvent for the first time, which were about 100 nm in diameter and composed of very small nanoparticles. The as-prepared samples were characterized using X-ray diffraction, scanning electron microscopy, and transmission electron microscopy. The results showed that the product was α-Fe_2_O_3_ nanosphere, and the temperature was an important factor on the formation of α-Fe_2_O_3_ nanospheres.

## Background

In the past decade, iron oxides have attracted an enormous amount of interest because of their great scientific and technological importance in catalysts, pigments, and gas sensors [1-3]. Among these iron oxides, α-Fe_2_O_3_, which is the most stable iron oxide with n-type semiconducting properties under ambient conditions, is the most researched and most frequently polymorphed in nature as the mineral hematite. Hematite has a rhombohedrally centered hexagonal structure of the corundum type with a close-packed oxygen lattice in which two-thirds of the octahedral sites are occupied by Fe^3+^ ions [4]. Recently, a lot of researches have been carried out on α-Fe_2_O_3_ due to its low cost and nontoxic property as an anode material for lithium-ion secondary batteries [5-7]. In fact, all researches have almost focused on the preparation of α-Fe_2_O_3_ nanostructured materials, because nanoscale materials often exhibit physical and chemical properties that differ greatly from their bulk counterparts. Various α-Fe_2_O_3_ with nanostructures have been prepared, such as nanoparticles [5,8-10], nanorods [11], nanotubes [12], flower-like structures [13], hollow spheres [14], nanowall arrays [15], dendrites [16], thin film [17,18], and nanocomposites [19-21].

In this work, we report one-pot method to prepare α-Fe_2_O_3_ nanospheres by solvothermal method using 2-butanone and water mixture solvent for the first time. The product is α-Fe_2_O_3_ nanosphere with an average diameter of approximately 100 nm, which is composed of a lot of very small nanoparticles. The temperature takes an important influence on the formation of α-Fe_2_O_3_ nanospheres.

## Methods

In a typical experimental synthesis, 0.1 g of Fe(NO_3_)_3_∙9H_2_O (≥ 99.0%) was dissolved in 3 mL of deionized H_2_O under stirring. Then, 37 mL of 2-butanone was added to the above solution. The mixture was stirred for about 10 min and then was sealed in a Teflon-lined stainless steel autoclave (50-mL capacity). The autoclave was maintained in an oven at 140°C for 12 h. The crude product was washed with anhydrous ethanol three times and finally dried in a vacuum chamber at 60°C for 10 h. The products were characterized by powder X-ray diffraction (XRD) performed on a Philips X'Pert diffractometer (Amsterdam, Netherlands) with CuK*α* radiation (*λ* = 1.54178 Ǻ). Scanning electron microscopy (SEM) images were taken on a JEOL JSM-6700F scanning electron microscope (Tokyo, Japan). Transmission electron microscopy (TEM) images and high-resolution TEM (HRTEM) images were obtained on the JEOL-2010 transmission electron microscope operating at 200 kV. The corresponding selected area electron diffraction (SAED) patterns were taken on a JEOL 2010 high-resolution TEM performing at 200 kV. The samples used for SEM, TEM, and HRTEM characterization were dispersed in absolute ethanol and were slightly ultrasonicated before observation.

## Results and discussion

**Figure 1:**
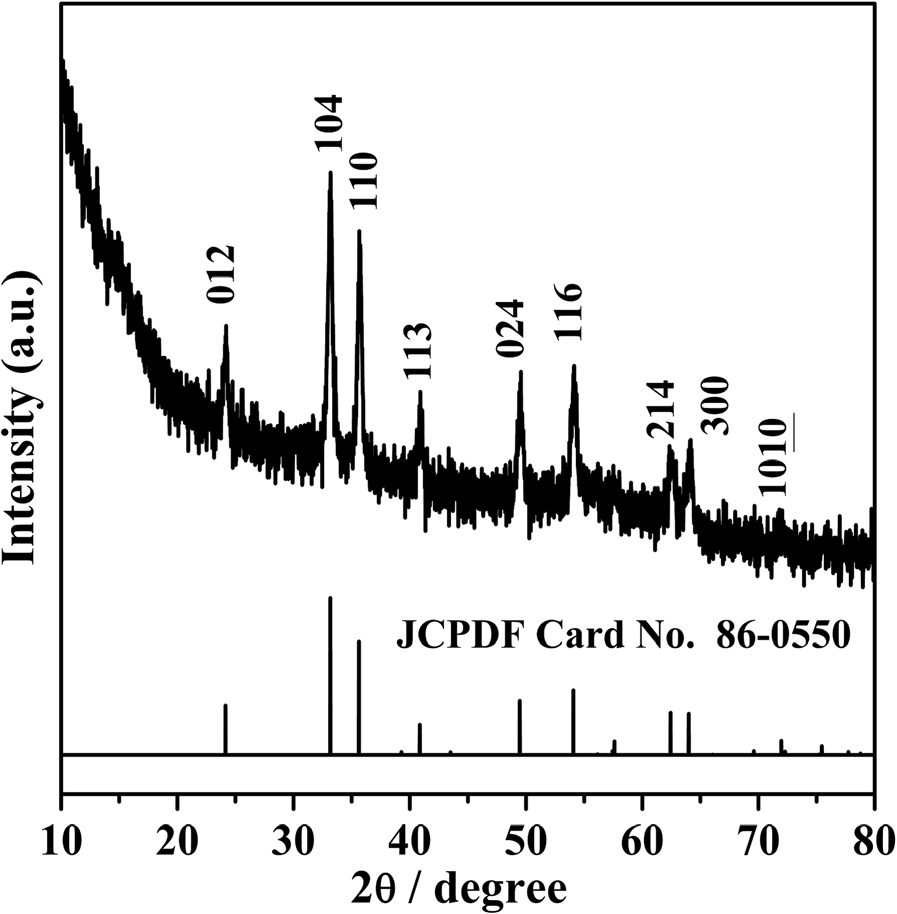
XRD pattern of a typical sample.

The phase purity of the product was examined by X-ray diffraction. Figure 1 shows the XRD pattern of a typical sample. All peaks can be indexed to the standard rhombohedral hexagonal phase Fe_2_O_3_ (JCPDF Card No.86-0550 $\bar{3}c$) and there are no additional peaks of impurities, indicating that it is pure α-Fe_2_O_3_.

**Figure 2:**
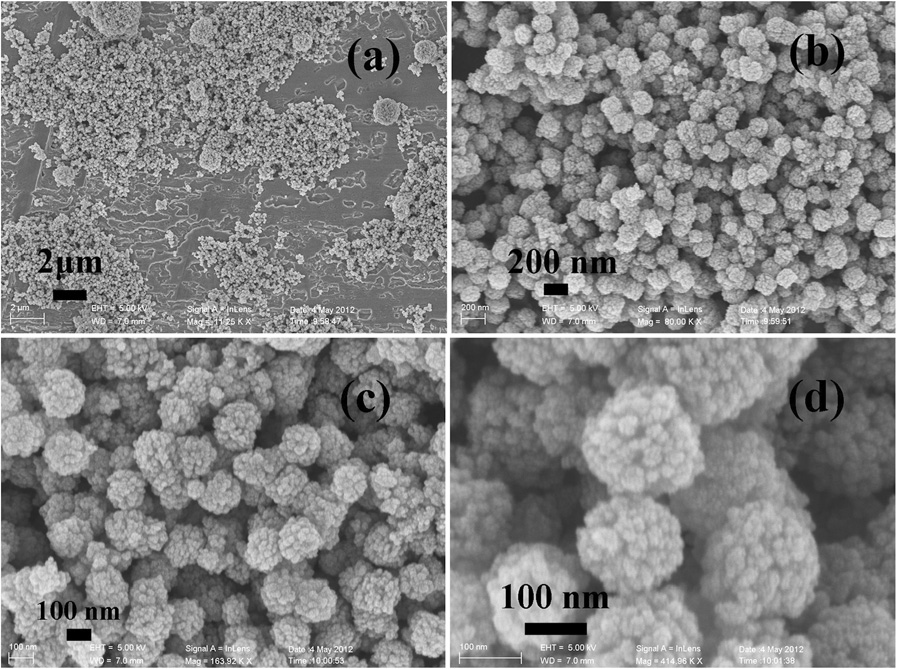
**SEM images of the product obtained in a typical synthesis.** (**a**-**b**) Low magnification, (**c**-**d**) high magnification.

**Figure 3:**
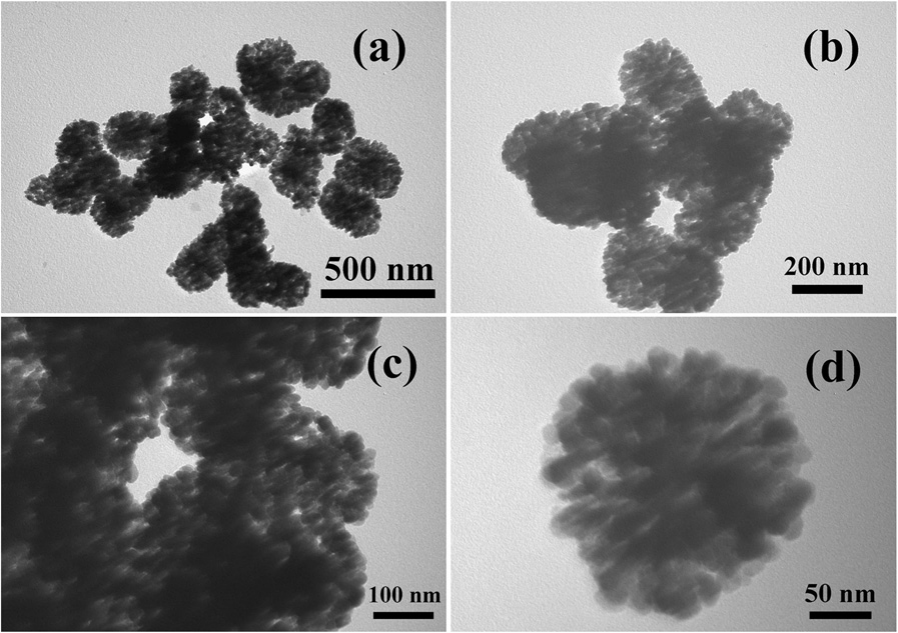
**TEM images of the product obtained in a typical synthesis.** (**a**-**b**) Low magnification, (**c**-**d**) high magnification.

The morphologies and microstructures of the typical sample have been studied by SEM and TEM. The SEM images (Figure 2) show that the product consists of well-dispersed spheres with a coarse surface. In the high magnification SEM image (Figure 2c, d), a great number of cracks on the surface of the spheres can be clearly observed, indicating the porous structure of the spheres with a diameter about 100 nm. In fact, every one sphere is composed of various smaller nanoparticles. The low and high magnification TEM images (Figure 3) also reveal that a lot of very small nanoparticles are loosely assembled to the nanosphere with an average diameter of about 100 nm, resulting into many gaps in these spheres. In other words, the SEM and TEM images together conform that the as-synthesized products are uniform nanospheres.

**Figure 4:**
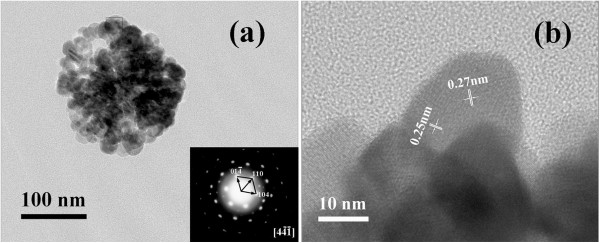
**Image of a single sphere.** (**a**) TEM image and (**b**) HRTEM image. Inset shows the corresponding SAED image from the marked part in (**a**).

To further investigate the particular structure of the α-Fe_2_O_3_ nanospheres, the HRTEM images of the typical sample are demonstrated in Figure 4. It can be clearly observed that a lot of gaps exist in the product, and the average diameter of the nanoparticles is about 25 nm (Figure 4a). In fact, we can estimate the size of the crystalline grains by Scherrer formula as well. Based on the typical reflection of the (104) crystalline plane (Figure 1), the crystallite size was calculated to be about 27 nm. Obviously, the two results are almost the same. The HRTEM image shows resolved lattice fringes of (104) and (110) planes with a spacing of almost 0.27 and 0.25 nm (Figure 4b), consistent with the XRD results. The inset in Figure 4a shows the SAED pattern taken from the marked part, which can be indexed to a rhombohedral hexagonal phase (space group $\bar{3}c$) with lattice constants *a* = 0.5035 nm and *c* = 1.3747 nm.

**Figure 5:**
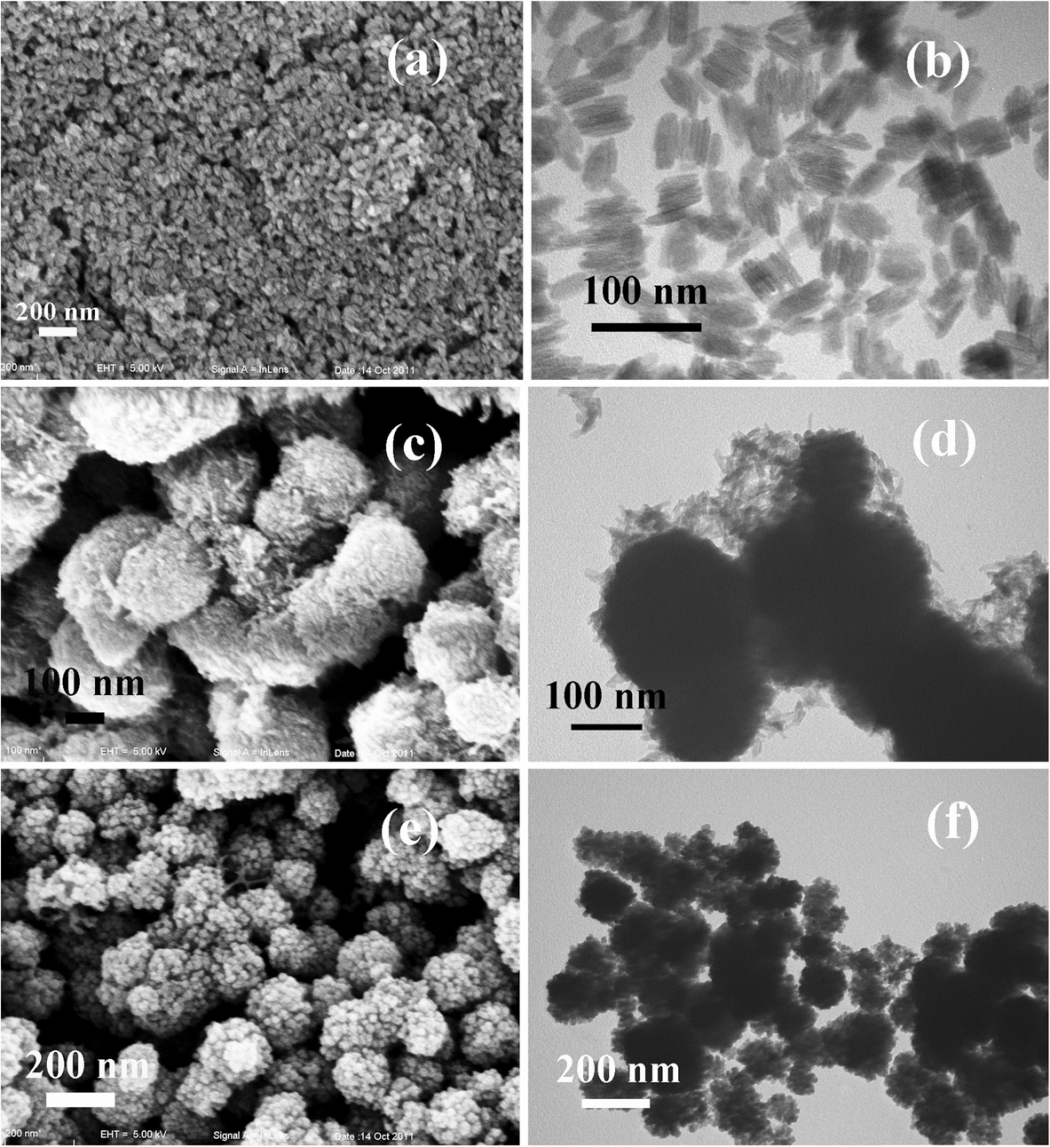
**SEM and TEM images of the products obtained at different reaction temperatures.** (**a**-**b**) 80°C, (**c**-**d**) 100°C, (**e**-**f**) 120°C. Other conditions were the same as those in the typical synthesis.

Moreover, the influence of reaction temperature on the product was investigated. Temperature plays a crucial role in the formation of well-defined spherical product. For example, keeping other experimental conditions the same with the typical synthesis when the temperature was reduced from 120°C to 80°C, significant morphology change was observed, which is shown in Figure 5. At 80°C, the obtained product was a nanorod (Figure 5a, b), which was FeOOH, similar to the previous work [22]. When the temperature was 100°C, the nanospheres were obtained (Figure 5c, d). However, under careful survey, we could find that the nanospheres were composed of many FeOOH nanorods. Increasing the reaction temperature to 120°C, the morphologies of the product (Figure 5e, f) were almost the same with the product in the typical synthesis except the inferior perfection.

## Conclusions

In conclusion, we have successfully prepared α-Fe_2_O_3_ nanospheres by solvothermal method using 2-butanone and water mixture solvent for the first time, which are about 100 nm in diameter and are composed of very small Fe_2_O_3_ nanoparticles. The temperature takes an important influence on the formation of the α-Fe_2_O_3_ nanospheres. The as-fabricated α-Fe_2_O_3_ nanospheres are expected to be applied in nanocatalysts, nanosensors, and lithium-ion secondary batteries.

## Competing interests

The authors declare that they have no competing interests.

## Authors’ contributions

CW prepared the manuscript and carried out the experiment. KT helped in the technical support for the characterizations. YC participated in the experiment. All the authors discussed the results and read and approved the final manuscript.

## Authors’ information

CW got his PhD degree in 2012. He has devoted his effort in the research of two- and three-dimensional new materials for several years. His research interests focused on the fabrication and application of two and three-dimensional new materials. He has published his works in several important international journals. KT has main interest in superconductivity with high-temperature superconductors. YC mainly researches the preparation of new catalysts.
